# Construction and validation of a scale of sexual self-concept for the elderly Chilean population

**DOI:** 10.12688/f1000research.122077.2

**Published:** 2023-11-20

**Authors:** Mauricio Ramirez-Perez, Rodrigo Ferrer-Urbina, Angela Flores, Valerie Garcia, Michelle Llancabure

**Affiliations:** 1 Direccion de Investigación, Postgrado y Transferencia Tecnológica, Universidad de Tarapaca, Arica, 100000, Chile; 2 Departamento de Psicología y Filosofia, Universidad de Tarapacá, Arica, 1000000, Chile

**Keywords:** sexual self-concept, elderly, scale, sexuality

## Abstract

Background: Sexual self-concept has a central role in the life of the elderly population. Indeed, their sexual self-concept has significant and positive relationships with their satisfaction with life, pleasure, and willingness to interact with others. However, social-cultural prejudice means that the elderly are considered asexual individuals, harming their sexual self-concept. This prejudice is prominent in Chile, where the elderly do not have access to clear information about their sexuality. However, research on the Chilean elderly population is still in its infancy and requires more attention. Hence, this research aims to construct and validate a scale of sexual self-concept for the elderly Chilean population to cover this identified gap in the literature. Methods: Sixty items were integrated into the first version of the scale. Ten external judges were asked to assess the content validity. Twenty-eight items were maintained. Subsequently, an instrumental and cross-sectional design was implemented with a non-probabilistic sampling (N = 188). Items were refined with corrected homogeneity indices and conditional estimates of Cronbach’s alpha and Omega coefficient. Results: A final scale of nine items equally distributed in three dimensions was obtained: Sexual self-efficacy (ω = 0.867), Sexual assertiveness (ω = 0.764), and Sexual self-esteem (ω = 0.803). The confirmatory factor analysis reflects that the theoretical model has an adequate fit (CFI = .989; TLI = .984; RMSEA = .086). Conclusions: The data analyses confirmed that the scale has adequate psychometric properties. This scale can be used for multidimensional measurements of sexual self-concept in the elderly in Chile. Further research can confirm its psychometric properties in different settings within the Spanish language population.

## Introduction

The literature has proposed that people’s lifestyles vary according to their cultural backgrounds ( Leung & Cohen, 2011; van Eijck & Bargeman, 2004). However, there is an acknowledgment that many societies have common themes such as human rights, democracy, mutual respect, social responsibilities, and achieving quality of life ( Deacon *et al.*, 2006). In this line of knowledge, sexuality is also progressively being identified as contributing to a fulfilling life ( Spong, 1988). Indeed, according to the World Health Organisation (2006), sexuality is a core component in developing the life cycle of individuals and their wellbeing.

Sexuality is a broad concept that integrates gender, identity, sexual desire, pleasure, eroticism, intimacy, sexual orientation and reproduction ( Morley & Kaiser, 2003). Sexuality is not exclusively for younger individuals, nor should it be ( Weeks, 2002). Indeed, sexuality concerns as much the elderly as it does younger individuals ( McAuliffe *et al.*, 2007). For the elderly, sexuality should be a normal and healthy activity ( Papalia & Martorell, 2017). Research has suggested that for the elderly, concepts such as life satisfaction, psychological wellbeing and cognitive functioning have a positive relationship with their sexuality ( Trudel *et al.*, 2008).

Previous research has identified that the elderly face two potential challenges to their sexuality ( Perdomo *et al.*, 2013). First, this age group frequently have associated issues with their sexuality, including sexual dysfunction and low sexual desire ( Camacho & Reyes-Ortiz, 2005). Second, the prevalence of socio-cultural prejudices and mythical beliefs about age negatively impact the development of sexuality at this stage ( Benbow & Jagus, 2002; Bouman *et al.*, 2006; Gázquez *et al.*, 2009; Lopes de Alencar *et al.*, 2014; Merghati-Khoei *et al.*, 2016). Based on socio-cultural prejudices, individuals from this age group are considered highly inhibited or sexually inactive ( Hodson & Skeen, 1994), sexually undesirable, unable of having a sexual life, or asexual individuals ( Di Napoli *et al.*, 2013).

These socio-cultural prejudices are common in Chile because the elderly do not have access to clear information about their sexuality ( Arnold *et al.*, 2007). According to the Observatorio del Envejecimiento (2021), Chile is one of the countries with high levels of prejudice against the elderly worldwide. As a result, ageism has been established in daily life in Chilean society. Ageism is defined as stereotypes, prejudice or discrimination against the elderly based on their age or perception of them as old individuals ( Luo *et al.*, 2021). Ageism includes considering the elderly as older to have a sexual life ( Archibald, 2003). It implies that Chilean society rejects sexuality in the elderly ( Herrera, 2003), implying that the elderly are asexual individuals ( Brigeiro, 2006).

The literature has proposed that evolutionary and disengagement theories are the roots for considering the elderly as asexual individuals ( Sharpe, 2004). First, the evolutionary theory proposes that sex has a functional purpose: procreation ( Róisín, 2013). Thus, because the elderly are not in their fertile period they are not considered sexual individuals. Secondly, the disengagement theory proposes that both society and individuals reciprocally participate in a gradual withdrawal from each other later in life ( Cumming & Henry, 1961). This theory suggests that elderly individuals change their daily lives from active to passive voluntarily ( Róisín, 2013). Therefore, the asexuality of the elderly is inferred from this theory because asexuality is associated with social passivity. At any rate, considering the elderly as asexual individuals is a misconception that harms their sexual self-concept ( Weeks, 2002).

Sexual self-concept has been proposed to be an active and dynamic structure built by organised perceptions of own sexual features into a cohesive construct ( Deutsch *et al.*, 2014). Furthermore, sexual self-concept is determined by the integrative accumulation of both external and internal information, which is judged and valued ( González *et al.*, 1997). Therefore, it is a cognitive view of sexual aspects of oneself derived from previous experience and manifested in recent experience, which can influence the processing of sexually relevant social information and guide individuals’ sexual behaviour ( Andersen, 1999; Andersen & Cyranowski, 1994). In the literature, there is an agreement that sexual self-concept is a multidimensional concept integrated by lower-order factors ( Deutsch *et al.*, 2014).

In particular, it has been proposed that self-concept in the elderly is integrated by three lower-order factors: sexual self-efficacy, sexual assertiveness and sexual self-esteem ( Snell, 1988). Sexual self-efficacy is defined as individuals’ perceived control of or confidence in the capability to achieve a given sexual outcome ( Closson *et al.*, 2018). This concept is considered relevant to keeping a satisfying and healthy sexual life ( Zimmer-Gembeck, 2013). Sexual assertiveness is proposed to be integrated by abilities related to initiating and communicating about desired sexual intercourse, refusing unwanted sexual intercourse, and communicating about sexual history and risk ( Loshek & Terrell, 2014). Therefore, individuals with high levels of sexual assertiveness are able to meet their sexual needs ( Sayyadi *et al.*, 2019). Finally, sexual self-esteem has been conceptualised as individuals’ affective response to the subjective appraisals of personal sexual thoughts, feelings, and behaviour ( Zeanah & Schwarz, 1996). In other words, sexual self-esteem would be the worth one gives to oneself as a sexual individual. Sexual self-esteem has a positive relationship with satisfaction with life, pleasure, willingness to interact with others and the possibility to develop intimate relationships ( Mayers *et al.*, 2003).

International research has proposed several scales to assess the self-concept in the elderly, including women’s sexual self-schema ( Andersen & Cyranowski, 1994); the self-concept scale ( O’Sullivan *et al.*, 2006; Reid *et al.*, 1977); multidimensional sexual scale ( Snell, 1988); and the sexual self-esteem inventory for women ( Zeanah & Schwarz, 1996), which among others have been demonstrated to be reliable and consistent. Notwithstanding that in Chile, the elderly are the age group with the most demographic growth ( Instituto Nacional de Estadística, 2017), research about their sexuality is scarce and requires more attention ( Herrera, 2003; Reyes & Castillo, 2011). In particular, there are no available validated scales to assess the sexual self-concept in the elderly for the local population.

This research aims to fill this gap in Chilean elderly research by developing and validating a measure of the concept. The option to validate a previous international scale was not considered for two related issues. First, the translation from the original language to another represents a methodological problem ( Hachey *et al.*, 1995). Second, the cultural representation of the original measure might be not a good representation in a different cultural setting ( Ramada *et al.*, 2013). These related issues are based on the quality of the translation and the comparability of different cultural or ethnic populations ( Sperber, 2004). Consequently, it was decided to develop a valid scale to evaluate the sexual self-concept of the elderly within the Chilean population to address the identified gap.

## Methods

### Stage 1: Generating and judging measurement items

After a literature review, the first version of the scale was designed to assess elderly sexual self-concept. Sixty items integrated this version of the scale. In order to further assess the content aspects of content validity, the items were rated by ten external expert judges ( Netemeyer *et al.*, 2003) who are psychologists with knowledge in areas of human development and instrument validation and were independent of those who developed the items ( Boateng *et al.*, 2018). The expert judges were presented with all the items alongside definitions of the three dimensions of the scale: sexual self-efficacy, sexual assertiveness, and sexual self-esteem. They were asked to (a) assign each item to whichever component they felt most relevant, and (b) rate each item from 1 (not adequate) to 3 (clearly adequate) in terms of how well they felt it represented the scales that they had selected. Furthermore, the expert judges were asked to provide qualitative feedback on item readability, wording, clarity, and overlap. Items were maintained if classified in the original scale and rated as clearly representative unanimously by the expert judges. As a result, 28 items were maintained (sexual self-efficacy = 9; sexual assertiveness = 9 and sexual self-esteem = 10).

### Stage 2: Internal reliability and factor analysis

**Study design**

This research was conducted following an instrumental and cross-sectional design. We initially intended to use a probabilistic design. However, it was impossible due to three main issues. Firstly, an elderly national registration is not available to allow a probabilistic design. Secondly, a considerable number of potential participants refused to answer about their sexual life. Finally, many potential participants were excluded due to their difficulties in completing the scale’s items, including literacy, poor item comprehension, and visual issues. Hence, a non-probabilistic design was implemented.

**Participants**

Participation in the study was voluntary and written informed consent was obtained from the study participants. Furthermore, participants were not asked to provide any personal or identifying information, such as names, phone numbers or addresses, were requested from participants. After that, they were asked to complete a paper survey of the designed scale and demographic information. As a result, a sample compromised by 223 elder participants was achieved using convenience sampling. This technique is the most used sampling in social sciences; members of the target population (e.g., Chilean elderly) that meet practical criteria (e.g., availability) are included to achieve research aims ( Burton *et al.*, 2018).

**Data collection**

A self-report survey was chosen as an appropriate methodology to conduct this study ( Liamputtong, 2017). Self-report surveys help identify and describe participants’ psychological features from their own perspectives, including subjective experiences, such as the variables involved in this study ( Paulhus & Vazire, 2007). Notwithstanding that, an online survey methodology offers benefits, including low cost, ease of distribution of multiple measures and speed of data gathering ( Buchanan & Hvizdak, 2009; Das *et al.*, 2018) it was not considered. The elderly usually have little access to the internet or computers and lack computer literacy ( Wang & Tang, 2021), representing issues for this age group to adequately complete an online survey.

Announcements in Universidad de Tarapacá, Arica -Chile were posted to invite students’ family members, relatives, or friends to meet the research criteria (individuals aged over 65 years old, which are considered elderly in the country) to participate in this study. The data collection was taken between June and September 2021. A paper-based survey was distributed to the participants. All the data was collected in a classroom of the Universidad de Tarapacá.

The initial sample was compromised of 223 elder participants. After a review of their answers, 23 participants failed in attention items. Furthermore, 12 participants do not answer five or more items. These 35 participants were discarded from the total sample. Thus, the final sample was compromised of 188 elder participants with more prevalence of females (N = 145, 77.1%) than males (N = 43, 22.9%), and a mean age of 72.43 years old (SD = 6.17). Participants were asked to answer the 28-item version of the scale (sexual self-efficacy = 9; sexual assertiveness = 9 and sexual self-esteem = 10).

**Ethics and consent**

This study was approved by the Scientific Ethical Committee of the Universidad de Tarapacá (approval no. 29/2017). Informed, written consent was obtained from participants. Participants were informed about the research objectives, and it was established that participation in the study was voluntary. Furthermore, participants were also informed that they could withdraw from the research at any stage.

**Statistical analyses**

First, the 28-item version of the scale was refined through item analysis (corrected homogeneity indices) and conditional estimates of reliability coefficients (Cronbach’s alpha and Omega coefficient). The analyses were carried out through Mplus 7.4 and JASP 0.9. As a result, the final scale to assess elderly sexual self-concept is integrated by nine items equally distributed in three dimensions: sexual self-efficacy, sexual assertiveness, and sexual self-esteem. Second, a confirmatory factorial analysis was carried out, following recommendations on the factorial treatment of ordinal variables based on the matrix of polychoric variables ( Garrido *et al.*, 2011). The robust weighted least estimation method squares (WLSMV) were selected, which are robust with non-normal discrete variables ( Asparouhouv & Muthén, 2007).

## Results

**Figure 1.**
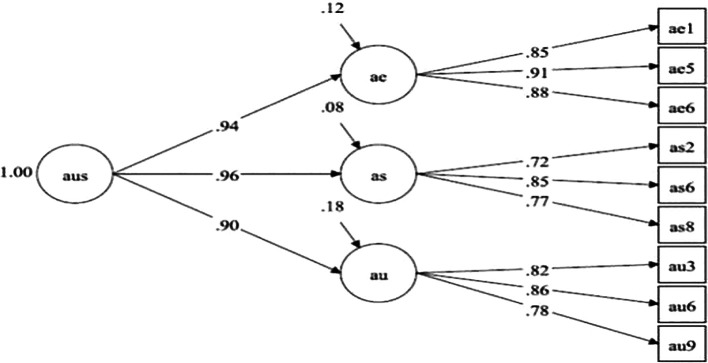
Representation of second-order model. Note: aus = sexual self-concept; ae = sexual self-efficacy; as = sexual assertiveness; au = sexual self-esteem.

Table 1 presents the fit of the rectified measurement model. Items were excluded for factor loadings less than.4, not being statistically significant or excessive redundancies between items. This process implied the elimination of 19 items. The final model is represented as a second-order model ( Figure 1). Notwithstanding some indicators of relative fit (RMSEA > .06) reflect a slight deviation from the standards recommended ( Schreiber *et al.*, 2006), which is expected given the small sample size, the incremental fit indicators (e.g., CFI > .95; TLI > .95) indicate that the model is an adequate population representation of the observed relationships ( Schreiber *et al.*, 2006).

**Table 1.** Model fits statistics.

N° Par	X ^2^	GL	P	CFI	TLI	RMSEA	RMSEA (IC 90%)	
							Inf	Sup
48	57.054	24	.0002	.989	.984	.086	.057	.114

Table 2 shows the standardised factor loadings for each dimension and the reliability estimates for each dimension. The items are presented in the original language of the scale, Spanish. After, the items are presented in English language for better comprehension of it. The full raw data from the study can be found under *Underlying data* ( Ramirez-Perez, 2022).

**Table 2.** Standardised loadings and reliability estimates by dimensions.

Item	Sexual self-efficacy	Sexual assertiveness	Sexual self-esteem
1 Puedo satisfacer mis deseos sexual	.855		
2 Puedo tener relaciones sexual	.905		
3 Podría ser un/a buen/a compañero/a sexual	.884		
4 Comunico mis deseos sexual		.718	
5 Manifiesto mi interés en tener relaciones sexuales con mi pareja		.852	
6 Me siento cómodo/a hablando de sexo con mi pareja		.773	
7 Siento que soy sexualmente deseable			.822
8 Me siento a gusto con mi cuerpo durante el sexo			.857
9 Me siento seguro/a al empezar una relación sexual			.779
α	.866	.764	.799
ω	.867	.764	.803

Items translated into English:

1. I can satisfy my sexual desires
2. I can have sexual relationships
3. I might be a good sexual partner
4. I can communicate my sexual desires
5. I can express my interest in having sexual relations with my partner
6. I feel comfortable talking about sex with my partner
7. I feel that I am sexually desirable
8. I feel comfortable with my body during sex
9. I feel safe when starting a sexual relationship

## Discussion

The right to sexual health during throughout one’s life is increasingly being considered a human right and a central part of a healthy life ( Merghati-Khoei *et al.*, 2016). However, based on socio-cultural prejudices, the elderly are often considered asexual individuals and therefore aren’t given this right, impacting their sexual self-concept. Individuals holding a negative valorisation of their sexual self-concept see themselves as conservative, less passionate, embarrassed, less sexual and often have low levels of sexual self-esteem ( Andersen, 1999; Cyranowski & Andersen, 1998). Furthermore, it has been proposed that low sexual self-concept has a positive relationship with depression symptoms ( Heidari *et al.*, 2017). To reverse this situation is necessary in order to measure sexual self-concept in the elderly correctly. However, as mentioned earlier, there is no valid scale to explore this concept within the Chilean elderly population.

Therefore, this research aimed to develop a valid measure of sexual self-concept in the elderly to fill this gap in the Chilean elderly research. At the beginning of the research, sixty items were considered in the scale to be included in the identified three principal dimensions of the sexual self-concept in the elderly: sexual self-efficacy, sexual assertiveness and sexual self-esteem. After a revision by ten external judges with knowledge in areas of human development and instrument validation, 32 items were deleted based on content analysis. This analysis refers to the adequacy with which a scale evaluate a particular area on interest correctly ( Hinkin, 1995). The requirement to have content validity is crucial if the items are to measure what they are presumed to measure ( DeVellis & Thorpe, 2021). Moreover, this validity establish content relevance and representations ( McPhail, 2007), i.e., that the items reflect the relevant experience of the selected population being examined. According to Boateng *et al.* (2018), content validity is mostly assessed through evaluation by expert or external judges, who are highly knowledgeable about the topic of interest and scale development. It has been suggested to have multiple judges (ranging from 5 to 7) to evaluate each of the items to determine whether they represent the domain of interest ( Haynes *et al.*, 1995). Therefore, this procedure was a reasonable decision and in agreement with previous discussion about it.

With the refinement of the scale, through item analysis (corrected homogeneity indices) and conditional estimates of reliability coefficients (Cronbach’s alpha and Omega coefficient) 19 items were deleted. These type of analyses are used frequently to assess and eliminate items that do not correlate strongly with the assessed construct ( Clark & Watson, 2019). Item analysis is a process which examines participants’ response to individual test items in order to assess the quality of those items and of the scale as a whole ( Downing, 2010). Furthermore, Cronbach’s alpha was developed to provide a measure of the internal consistency of a test or scale (Cronbach, 1951). Internal consistency describes how closely related a set of items are as a group ( Tavakol & Dennick, 2011). Finally, the Omega coefficient was considered because it provides an overall assessment of reliability. Omega coefficients provide detailed outputs about the reliability of measures and are less likely to be misinterpreted because of the transparent relationship between coefficients ( Green & Yang, 2015). Therefore, the selected analyses were useful to refine the scale. Finally, the remaining nine items were equally distributed into the three factors of the scale. The statistical results showed that this scale has adequate psychometric properties, and the model is an adequate population representation of the observed relationships.

The relevance of this research is to build and validate an original scale of sexual self-concept in the elderly in the Spanish language and by considering the local cultural representation. It is a short one-dimensional short scale (9 items) that were considered. This scale can be used for research purposes and to understand better sexual self-concept in the elderly in Chile. Furthermore, applying this scale in different settings and participants can provide further insights into its psychometric properties.

### Limitations

This research has limitations to be noted. It was explained earlier that three main issues were faced to achieve a probabilistic design. First, notwithstanding that the approximate population of the elderly is 2.2 million, representing 11.9% of the Chilean population ( Instituto Nacional de Estadística, 2019), an elderly national registration is not available. This limitation offers further policy implications. Complete registration of the Chilean elderly might help identify the care and support needed for them. Furthermore, it will facilitate future research and interventions for this age group.

Second, due to the research topic, a considerable number of potential participants refused to participate in the study. Sexuality is a sensitive research topic that implies that participants reveal personal information ( Kuyper *et al.*, 2012). To address this limitation, further research on sexuality could avoid this issue by using a larger sample of participants.

A third issue was that potential participants with literacy issues, poor item comprehension and visual issues were excluded from the final sample. These issues are common in research among the elderly; this age group can lose literacy skills, have less comprehension and have physical issues (visual and hearing) ( McHorney, 1996). Further research might consider visual elements to facilitate elderly responses, including soft colours and symmetrical and straightforward shapes in their questionnaires ( Jones *et al.*, 2013).

Finally, most of the participants are from one Chilean city (Arica). Thus, applying this scale in different settings is recommended, so that a wider sample of participants can deliver further insights into its psychometric properties.

## Conclusions

The relevance of this research is to build and validate an original scale of sexual self-concept in the elderly in the Spanish language by considering the Chilean local representation. It is a brief scale integrated by nine items equally distributed in three dimensions: sexual self-efficacy, sexual assertiveness and sexual self-esteem. This scale can be used for research purposes and to understand better the sexual self-concept in the Chilean elderly population.

## Data availability

### Underlying data

OSF: Construction and validation of a scale of sexual self-concept for the elderly Chilean population. https://osf.io/j23vg ( Ramirez-Perez, 2022).

This project contains the following underlying data:

- English version scale.docx
- Original_Database_SexualSelfConcept_Elderly.sav
- Original scale.docx

### Extended data

This project contains the following extended data:

- English Participant Scale.docx
- Spanish Original Participant Scale.docx

Data are available under the terms of the Creative Commons Zero “No rights reserved” data waiver (CC0 1.0 Public domain dedication).
