# Supplementary material for: Construction and validation of a scale of sexual self-concept for the elderly Chilean population
Source: F1000Res. 2023 Nov 20;11:1056. Originally published 2022 Sep 15. [Version 2] doi: 10.12688/f1000research.122077.2 (PMC10784661; doi:10.12688/f1000research.122077.2)
Supplement: Peer review history

# Reviewer report: Reviewer response for version 2

**DOI:** 10.5256/f1000research.157878.r224033

**Published:** 2024-01-11

**Authors:** Mauricio Gonzales Arias

**Affiliations:** 1 Department of Psychology, Universidad de La Serena, La Serena, Chile

The new version of the article conforms to what was requested. The observations raised were overcome. I consider that the article can be accepted for indexing.

Is the rationale for developing the new method (or application) clearly explained?

Yes

Is the description of the method technically sound?

Yes

Are the conclusions about the method and its performance adequately supported by the findings presented in the article?

Partly

If any results are presented, are all the source data underlying the results available to ensure full reproducibility?

Yes

Are sufficient details provided to allow replication of the method development and its use by others?

Yes

Reviewer Expertise:

I am a professor of psychometrics and research methods. I research emotional processes and university education.

I confirm that I have read this submission and believe that I have an appropriate level of expertise to confirm that it is of an acceptable scientific standard.

# Reviewer report: Reviewer response for version 1

**DOI:** 10.5256/f1000research.134015.r150564

**Published:** 2023-06-02

**Authors:** Marcelo Leiva-Bianchi

**Affiliations:** 1 Faculty of Psychology, Laboratory of Methodology, Behavioural Sciences and Neuroscience, Universidad de Talca, Talca, Chile

Contextually relevant article to assess sexual self-concept in older adults from a new population. However, there are the following doubts:

 What is the scientific problem? What is the discussion regarding the dimensions of sexual self-concept? Why does this scale improve previous proposals?

 Why choose to design a new scale, despite the fact that there are others that evaluate the construct?

 Why was the new scale applied without applying any of the other existing scales? What could happen with concurrent validity?

 Why start by assessing the reliability of the new scale and then move on to its construct validity? What if a dimension turns out to be reliable, but not valid?

 What if the construct validity of a new scale is based on confirmatory factor analysis? What happens if there are more or fewer dimensions than those proposed in the confirmatory analysis, when compared with a parallel factor analysis?

 What if the information in Table 1 were reported in the text of the article?

Is the rationale for developing the new method (or application) clearly explained?

Yes

Is the description of the method technically sound?

Yes

Are the conclusions about the method and its performance adequately supported by the findings presented in the article?

Partly

If any results are presented, are all the source data underlying the results available to ensure full reproducibility?

Yes

Are sufficient details provided to allow replication of the method development and its use by others?

Yes

Reviewer Expertise:

Methodology and psychometrics

I confirm that I have read this submission and believe that I have an appropriate level of expertise to confirm that it is of an acceptable scientific standard.

# Reviewer report: Reviewer response for version 1

**DOI:** 10.5256/f1000research.134015.r155868

**Published:** 2022-12-06

**Authors:** Mauricio Gonzales Arias

**Affiliations:** 1 Department of Psychology, Universidad de La Serena, La Serena, Chile

The construction and validation of a sexual self-concept scale in Chilean older adults is reported. It is based on the importance of sexuality and its relationship with well-being in the elderly and the lack of studies and instruments that address this issue in Chile. In addition, the population of older adults is increasing in the country and there are cultural barriers to addressing this issue. The method used is relevant and adequately described. The items elaborated and those selected are finally relevant to the theoretical model exposed. The final instrument of 9 items is suitable for a population that has multiple difficulties in answering this type of instrument and requires brief instruments.

 However, the discussion is very poorly developed and the reasons why a large number of items were eliminated need to be deepened. The possibility that the model evaluated is not reproduced in a different sample is not analyzed and the steps to follow in the validation of the instrument are not identified.

Is the rationale for developing the new method (or application) clearly explained?

Yes

Is the description of the method technically sound?

Yes

Are the conclusions about the method and its performance adequately supported by the findings presented in the article?

Partly

If any results are presented, are all the source data underlying the results available to ensure full reproducibility?

Yes

Are sufficient details provided to allow replication of the method development and its use by others?

Yes

Reviewer Expertise:

I am a professor of psychometrics and research methods. I research emotional processes and university education.

I confirm that I have read this submission and believe that I have an appropriate level of expertise to confirm that it is of an acceptable scientific standard, however I have significant reservations, as outlined above.

# Response

**Published:** 2023-10-20

**Authors:** Mauricio Ramirez-Perez

**Affiliations:** Universidad de Tarapaca, Chile

Dear reviewer, 

 We appreciate your comments that help us to improve our article. Your comments were addressed. In particular, a more comprehensive explanations about the deleted items is provided. Moreover, an explanation about further use of this scale is delivered.

 Many thanks,

 Sincerly

 Mauricio R.
